# Correlation of Physical Exam Findings with Fever in Patients with Skin and Soft Tissue Infections

**DOI:** 10.5811/westjem.2016.12.32838

**Published:** 2017-02-27

**Authors:** Jillian Mongelluzzo, Brian Tu, Barbara Grimes, Sharvina Ziyeh, Jonathan Fortman, Jersey Neilson, Robert M. Rodriguez

**Affiliations:** * University of California, San Francisco, San Francisco General Hospital, Department of Emergency Medicine, San Francisco, California; † University of California, San Francisco, San Francisco, Department of Epidemiology and Biostatistics, San Francisco, California

## Abstract

**Introduction:**

The objectives of this study were to determine the prevalence of fever in adult ED patients with skin and soft tissue infections (SSTI) and to determine which, if any, physical exam, radiograph and laboratory test findings were associated with fever.

**Methods:**

We conducted a prospective, observational study at an urban county trauma center of adults who presented to the ED for evaluation of suspected SSTI. ED providers measured area of erythema and induration using a tape measure, and completed data sheets indicating comorbid conditions and the presence or absence of physical exam findings. Fever was defined as any recorded temperature ≥ 38°C during the first six hours of ED evaluation.

**Results:**

Of the 734 patients enrolled, 96 (13.1%) had fever. Physical and laboratory exam findings associated with the presence of a fever in multivariable logistic regression were the area of erythema, particularly the largest quartile of area of erythema, 144 – 5,000 cm^2^, (odd ratio [OR] = 2.9; 95% confidence interval [CI] [1.6 – 5.2]) and leukocytosis (OR = 4.4, 95% CI [2.7 – 7.0]). Bullae, necrosis, streaks, adenopathy, and bone involvement on imaging were not associated with fever.

**Conclusion:**

Fever is uncommon in patients presenting to the ED for evaluation of suspected SSTI. Area of erythema and leukocytosis were associated with fever and should be considered in future decision rules for the evaluation and treatment of SSTI.

## INTRODUCTION

Skin and soft tissue infections (SSTI), primarily cellulitis and abscesses, are increasingly common reasons for presentation to acute care facilities and admission to inpatient hospital facilities.^1^–^3^ Despite this commonality of SSTI, very little evidence-based literature addresses the early evaluation of SSTI, and acute care diagnosis and treatment are largely driven by traditional teaching and convention.

Medical decision-making in the emergency department (ED) is often based on the presence or absence of fever.^4^ The 2005 Infectious Disease Society of America (IDSA) guidelines acknowledge that the majority of SSTIs are not of significant severity but recommend further diagnostic evaluation in patients with signs and symptoms of systemic toxicity, including fever.^5^ The febrile patient with cellulitis or an abscess is much more likely to be admitted to the hospital than the afebrile patient. Sabbaj et. al. found that the presence of fever was the strongest predictor of need for hospitalization for > 24 hours.^6^ Similarly, it is standard practice to admit all injection drug users (IDUs) with fever and no clear source.^4^,^7^

Little is known regarding SSTI physical examination findings as they relate to fever. Lonergan et. al. reported that fewer than 25% of patients with necrotizing fasciitis have a fever on presentation.^8^ The objectives of this hypothesis-generating, prospective, observational study were to determine the prevalence of fever in patients presenting to the ED with SSTI and to determine which (if any) physical exam, laboratory and radiologic findings are associated with fever.

## METHODS

### Study design, setting and participants

This was an observational, prospective study of adult patients who presented to the ED of an urban county trauma center from June 2009 to January 2013. All patients greater than 18 years of age who presented to the ED for evaluation of suspected SSTI were eligible for enrollment. At the time of this research, the hospital had an urgent care clinic open on weekdays and a wound care clinic open two days per week; patients presenting primarily to these sites were not included.

We enrolled subjects according to the availability of research assistants, primarily from 11 a.m. to 11 p.m. on weekdays. Research staff and treating clinicians identified all potential enrollees based on chief complaints and treating-provider diagnosis of suspected SSTI. Patients were excluded if no temperature was recorded in the ED chart.

### Data collection

We recorded all temperatures measured in the initial six hours after ED presentation (including triage temperatures). Research assistants provided the data collection form to treating clinicians (attending physician, resident physician, or nurse practitioner), who recorded various elements of history (history of diabetes mellitus, known human immunodeficiency virus [HIV], reported history of injection drug use, prior trauma or surgery to area), physical exam (location of infection, area of erythema, presence or absence of purulence, bullae, adenopathy, streaks, necrosis), and laboratory (complete blood counts [CBC]) and imaging results (computerized tomography [CT] and plain film radiography). Treating clinicians were provided with a disposable paper centimeter ruler attached to the data collection form to measure the size of erythema and/or abscess, both recorded as total area (cm^2^).

Bone involvement on imaging was considered present if the patient had radiograph or CT imaging with evidence of osteomyelitis or periosteal reaction. A patient was considered to have multiple sites of infection if more than one area of the body had evidence of SSTI.

### Data Analysis

We primarily analyzed these data using standard descriptive statistics. We calculated odds ratios and 95% confidence intervals (CI) for the outcome of fever. Additionally, we performed tests of association (univariate analysis and logistic regression) to determine whether certain clinical findings, such as total area of erythema, were associated with fever. When analyzing CBC and imaging characteristics for association, we used only those patients who received those tests. We analyzed data using SAS (version 9.2; SAS Institute, Inc., Cary, NC).

**Population Health Research Capsule**

What do we already know about this issue?

Fever is a major factor in admission and treatment decisions in patients with skin and soft tissue infections (SSTI), especially in injection drug users (IDU).

What was the research question?

We sought to determine the prevalence of fever in patients presenting to the ED with SSTI and to determine patient characteristics associated with fever.

What was the major finding of the study?

Fever is uncommon in patients presenting to the ED with SSTI. Area of erythema and leukocytosis were the only characteristics associated with fever.

How does this improve population health?

Clinicians should consider these findings when considering admission and treatment decisions in patients with SSTI, especially in IDU.

## RESULTS

Of the 734 patients, 96 (13.1%; 95% CI [0.8–15.7]) had fever during the first six hours of ED evaluation. Their mean age was 45 years (interquartile range 35 – 55), 77%, were male, and 246 (33.5%) were admitted to the hospital with a hospital mortality of 0.6%. Current injection drug use was reported in 30% of patients; 18% had diabetes mellitus, and 8% were known to have HIV.

Febrile patients were more commonly admitted to the hospital (77.1% versus 27.3%; mean difference 49.8%: 95% CI [39.8%–57.8%]). The mortality of febrile patients (0.7%) was similar to that of those without fever (0.6%).

The mean total area of erythema in patients without a fever was 137 cm^2^ (standard deviation [SD] 378) and in patients with a fever was 187 cm^2^ (SD 294). In Tables 1 and 2 we present the univariate analysis, in which the characteristics of area of erythema, leukocytosis and adenopathy were associated with fever. However, when controlling for covariates in multivariable logistic regression (Table 3), only leukocytosis (odds ratio (OR) 4.4; 95% CI [2.7 – 7.0]; p < 0.0001) and higher quartiles of area of erythema remained statistically associated with fever.

## LIMITATIONS

Likely the greatest limitation of our study method was that we were unable to control for anti-pyretic use prior to or during ED evaluation, which may have decreased the rate of fever detected in our study. Although we captured all temperatures recorded by ED personnel in the first six hours, we may have missed spikes in temperature that were not observed by staff. We did not characterize other makers of systemic illness, such as tachycardia and tachypnea, which are also important data points in triage and treatment decisions for patients with SSTI. Finally, our ED demographics may not reflect the patients seen at other institutions, especially with regard to our large proportion of IDU patients and overall high admission rate of patients with SSTI. Many patients with minor SSTI were seen at the urgent care and wound care clinics – this spectrum bias would likely lead to an even lower rate of fever in patients with SSTI.

## DISCUSSION

Emergency medicine practitioners commonly treat patients with SSTI, and the presence or absence of fever is a major factor in admission and treatment decisions. In this prospective, observational study, we have characterized the presentations of patients with SSTIs with several findings that may impact their evaluation and management. First, we found that fever is relatively uncommon in patients with SSTIs – even in IDUs. This simple observation of prevalence of fever may be important when considering admission decisions, especially regarding the current standard practice of admitting febrile IDUs without a clear source. As expected, patients with fever were more commonly admitted to the hospital than afebrile patients. Both febrile and afebrile patient groups had very low overall mortality.

Second, we determined that area of erythema and leukocytosis are the only two clinical and laboratory characteristics associated with fever. When the area of erythema was less than 9 cm^2^, fever was unusual. IDUs commonly have small areas of erythema and abscess at injection sites. When evaluating IDUs with fever in the ED, practitioners should likely not attribute the fever to these minor areas of infection and should consider further evaluation and admission for the true source of fever.

Third, notable characteristics that have been stated to indicate more serious infections, such as bullae, streaks, necrosis and bone involvement on imaging, were not associated with fever. This finding is similar to that of other studies in which a minority of patients with necrotizing fasciitis and other serious infections had fever in the ED.^8^

Finally, the mortality for admitted patients was very low, precluding a meaningful analysis as to whether fever portends worse prognosis. Other investigators have similarly found low mortality rates among patients with SSTIs. Carratala et. al. found that the strongest predictor of mortality in patients with SSTIs was shock on presentation.^9^

Physical exam findings have been inconsistently used in prior studies to predict the need for hospitalization or outcomes in patients with SSTIs. In a study by Sabbaj et. al. the presence of fever was found to be a predictor of > 24-hour hospitalization in patients with non-facial SSTIs.^6^ The investigators attempted to create a clinical decision rule to guide hospital admission in patients with SSTIs, but were unable to create a highly sensitive model. Schrock et. al. conducted a study to predict those patients who would fail ED observation unit placement.^10^ The authors found that female patients and patients with white blood count greater than 15,000 to be more likely to require hospitalization, but other physical exam findings were not considered in the analysis.

In patients with the most severe form of SSTIs, necrotizing fasciitis, physical exam findings have also been inconsistently used in the diagnosis and the prediction of outcomes.^8^,^11^–^14^ The laboratory risk indicator for necrotizing fasciitis score (LRINEC), which has been shown to predict diagnosis and outcomes in patients with necrotizing fasciitis, includes leukocytosis in the score, but does not include any physical exam finding.^11^,^12^ Notably, fever has not been found to be a predictor of mortality or limb loss.^13^ However, in another study of patients with necrotizing infections, mean percentage of body surface area has been shown to be a predictor of mortality in patients.^14^

In the United States, ambulatory care visits and hospitalizations for SSTIs have been increasing.^1^,^3^ Given resource constraints and costs of inpatient care, appropriate disposition of patients with SSTIs is becoming increasingly important. Clinical decision rules for admission versus discharge and for triage to intensive care units have been developed for other illnesses, including infectious diseases like pneumonia.^15^,^16^ Considering the low mortality seen in SSTI patients, future investigations to similarly develop SSTI admission criteria will require multi-center protocols with larger sample sizes.

## CONCLUSION

Fever is uncommon in patients with SSTI. Of the physical and laboratory exam findings examined, only leukocytosis and area of erythema were associated with fever. Small areas of SSTIs are unlikely to be sources of fever in patients presenting to the ED. Mortality was very low in admitted patients with SSTI.

## Figures and Tables

**Table 1:** Univariate analysis of clinical characteristics (physical exam and laboratory findings) and fever in patients with SSTI.

	N (%)	Afebrile with finding %	Afebrile without finding %	Febrile with finding %	Febrile without finding %	OR (95% CI)	P-value
Purulence	430 (54%)	85%	87%	15%	13%	1.1 (0.8 – 1.8)	0.42
Adenopathy	143 (18%)	78%	87%	22%	14%	1.8 (1.1 – 2.8)	0.013
Bullae	81 (10%)	83%	86%	17%	14%	1.2 (0.7 – 2.3)	0.48
Multiple locations*	115 (14%)	81%	86%	19%	14%	1.5 (0.88 – 2.4)	0.15
Necrosis	37 (5%)	92%	85%	8%	15%	0.5 (0.2 – 1.7)	0.26
Streaks	116 (14%)	84%	86%	16%	14%	1.2 (0.7 – 2.0)	0.57
Leukocytosis (N = 601)	246 (41%)	69%	91%	31%	9%	4.4 (2.8 – 7.0)	<0.0001
Bone involvement on imaging (N = 406)	29 (7%)	83%	81%	17%	19%	0.9 (0.3 – 2.4)	0.81

*SSTI,* skin and soft tissue infection; *OR*, odds ratio.

* More than 1 SSTI location on individual patient

**Table 2:** Univariate analysis of area of erythema and fever in patients with SSTI.

Quartile	Presence of fever	OR (95% CI)	p-value
0.5 – 9 cm^2^	8%		
10 – 30 cm^2^	15%	2 (1.1 – 3.7)	0.026
32 – 140 cm^2^	15%	2 (1.1 – 3.8)	0.023
144 – 5000 cm^2^	20%	2.9 (1.6 – 5.2)	0.0004

*SSTI,* skin and soft tissue infection; *OR*, odds ratio.

**Table 3:** Multivariable logistic regression analysis of patient characteristics including physical exam findings and the presence of fever in patients with SSTI.

Variable	OR (95% CI)	p – value
Age	1.0 (0.9 – 1.0)	0.39
Purulence	1.2 (0.7 – 1.8)	0.53
Adenopathy	1.5 (0.9 – 2.5)	0.10
Multiple locations	1.4 (0.8 – 2.6)	0.22
Sex	0.8 (0.5 – 1.4)	0.55
Leukocytosis	4.4 (2.7 – 7.0)	<0.0001
Area of erythema		
Quartile 2	2.2 (1.1 – 4.4)	0.026
Quartile 3	1.9 (0.9 – 4.0)	0.058
Quartile 4	2.1 (1.034 – 4.1)	0.04

*SSTI,* skin and soft tissue infection; *OR*, odds ratio.

## References

[1] Pallin DJ, Egan DJ, Pelletier AJ (2008). Increased US emergency department visits for skin and soft tissue infections, and changes in antibiotic choices, during the emergence of community-associated methicillin-resistant Staphylococcus aureus. Ann Emerg Med.

[2] Hersh AL, Chambers HF, Maselli JH (2008). National trends in ambulatory visits and antibiotic prescribing for skin and soft-tissue infections. Arch Intern Med.

[3] Edelsberg J, Taneja C, Zervos M (2009). Trends in US hospital admissions for skin and soft tissue infections. Emerg Infect Dis.

[4] Rodriguez R, Alter H, Romero KL (2011). A pilot study to develop a prediction instrument for endocarditis in injection drug users admitted with fever. Am J Emerg Med.

[5] Stevens DL, Bisno AL, Chambers HF (2014). Practice guidelines for the diagnosis and management of skin and soft tissue infections: 2014 update by the Infectious Diseases Society of America. Clin Infect Dis.

[6] Sabbaj A, Jensen B, Browning MA (2009). Soft tissue infections and emergency department disposition: predicting the need for inpatient admission. Acad Emerg Med.

[7] Brown PD, Levine DP (2002). Infective endocarditis in the injection drug user. Infect Dis Clin North Am.

[8] Lonergan S, Rodriguez RM, Schaulis M (2004). A case series of patients with black tar heroin-associated necrotizing fasciitis. J Emerg Med.

[9] Carratala J, Roson B, Fernandez-Sabe N (2003). Factors associated with complications and mortality in adult patients hospitalized for infectious cellulitis. Eur J Clin Microbiol Infect Dis.

[10] Schrock JW, Laskey S, Cydulka RK (2008). Predicting observation unit treatment failures in patients with skin and soft tissue infections. Int J Emerg Med.

[11] Wong CH, Khin LW, Heng KS (2004). The LRINEC (Laboratory Risk Indicator for Necrotizing Fasciitis) score: a tool for distinguishing necrotizing fasciitis from other soft tissue infections. Crit Care Med.

[12] Su YC, Chen HW, Hong YC (2008). Laboratory risk indicator for necrotizing fasciitis score and the outcomes. ANZ J Surg.

[13] Anaya DA, McMahon K, Nathens AB (2005). Predictors of mortality and limb loss in necrotizing soft tissue infections. Arch Surg.

[14] Elliott DC, Kufera JA, Myers RA (1996). Necrotizing soft tissue infections. Risk factors for mortality and strategies for management. Ann Surg.

[15] Fine MJ, Auble TE, Yealy DM (1997). A prediction rule to identify low-risk patients with community-acquired pneumonia. N Engl J Med.

[16] Lim WS, van der Eerden MM, Laing R (2003). Defining community acquired pneumonia severity on presentation to hospital: an international derivation and validation study. Thorax.

